# CD226 Deficiency Attenuates Periodontal Bone Destruction in a Murine Ligature-Induced Periodontitis Model

**DOI:** 10.3390/biology15171483

**Published:** 2026-09-01

**Authors:** Yi Li, Wenjing Zhou, Xutao Chen, Wenli Dan, Lihua Chen, Fang Zhao, Liang Fang

**Affiliations:** 1 Department of Immunology, Air Force Medical University, Xi’an 710032, China; 2 Department of Occupational and Environmental Health, The Ministry of Education Key Lab of Hazard Assessment and Control in Special Operational Environment, The Shaanxi Provincial Key Laboratory of Environmental Health Hazard Assessment and Protection, The Shaanxi Provincial Key Laboratory of Free Radical Biology and Medicine, School of Public Health, Air Force Medical University, Xi’an 710032, China

**Keywords:** bone loss, CD226, osteoclast, periodontitis

## Abstract

Periodontal disease is widely recognised as a chronic infectious-inflammatory disorder, leading to the destruction of alveolar bone. Osteoclasts and their progenitors have been demonstrated to play pivotal roles in the process of periodontitis-induced bone damage. The mechanisms that govern the differentiation of osteoclasts in the context of periodontitis remain to be elucidated. In this study, we assessed the expression of CD226 on osteoclasts and their progenitors, as well as the function of CD226 in experimental periodontitis mice. Periodontitis was induced in C57BL/6 wild-type and CD226 knockout (CD226-KO) mice. Micro-CT was used to analyse bone loss. Haematoxylin–eosin (HE) or tartrate-resistant acid phosphatase (TRAP) staining was conducted to examine osteoclast differentiation. Flow cytometric analysis and quantitative real-time PCR (qRT-PCR) were used to identify the expression of CD226 and related molecules. The expression of CD226 on osteoclasts was found to decrease gradually during the process of osteoclastogenesis. In addition, the study revealed that the knockout of CD226 inhibited osteoclast differentiation in vitro. Furthermore, the knockout of CD226 has been demonstrated to attenuate inflammatory responses and alleviate bone damage in a murine periodontitis model. The present study suggests that CD226 plays an important role in regulating the function and differentiation of osteoclasts and their progenitors. The targeting of CD226 has the potential to be developed as a potent therapy for bone loss caused by periodontitis.

## Simple Summary

In this study, we found that CD226 is differentially expressed on osteoclasts and their progenitors in murine models, with its expression gradually decreasing during osteoclastogenesis. CD226 knockout inhibited osteoclast differentiation in vitro and attenuated inflammatory responses and bone damage in a murine periodontitis model. These findings suggest that CD226 plays a critical role in regulating the differentiation and function of osteoclasts and their progenitors, and targeting CD226 may offer a promising therapeutic approach for periodontitis-related bone loss.

## 1. Introduction

Periodontal disease is widely recognised as a chronic infectious inflammatory condition. It arises from a dysregulated host immune response to colonisation by dysbiotic subgingival microbial biofilms, which results in progressive destruction of periodontal supporting tissues and may ultimately lead to tooth loss. Among its clinical manifestations, alveolar bone loss represents the most prominent marker of disease severity and progression [1,2,3]. It is well established that the immune system contributes notably to the maintenance of bone homeostasis. The initial immune response in periodontitis is characterised by the infiltration of immune cells, which is followed by the activation of osteoclasts, resulting in an osteolytic inflammatory response and tissue destruction [1,2,4]. Lipopolysaccharide (LPS) derived from Gram-negative bacteria and host-derived interleukin-1 (IL-1) are well-established major drivers of inflammatory osteoclast activation. Recent studies have demonstrated that LPS predominantly triggers cytokine production via TLR4 signalling, including IL-1β, TNF-α, and IL-6. These cytokines amplify RANKL signalling to promote osteoclast differentiation. IL-1 further synergises with RANKL by accelerating osteoclast precursor maturation, enhancing multinucleation, and prolonging osteoclast survival [5,6,7]. Collectively, LPS-driven cytokine networks, together with IL-1 signalling, establish a strongly pro-resorptive microenvironment that underpins inflammatory bone loss.

Osteoclasts are defined as cells that mediate the resorption of bone tissues. They are derived from mononuclear myeloid hematopoietic stem cells located within bone marrow, and the fusion of numerous monocytes/macrophages initiates osteoclast formation [8,9,10,11]. As demonstrated in the previous study [12], adhesion molecules expressed on osteoclast precursors have been demonstrated to play key roles in osteoclastogenesis. These cell surface molecules play a pivotal role in either the interaction with other immune cells and stromal cells or the specific recognition and fusion among preosteoclasts. Consequently, a more profound comprehension of the expression and functions of these cell adhesion molecules in regulating osteoclast differentiation is imperative for the development of more efficacious therapies for bone loss in periodontitis.

CD226 (also known as DNAX-accessory molecule-1 (DNAM-1)) is a member of the immunoglobulin superfamily and acts as a cell adhesion molecule. It is implicated in various immunological processes and functions. CD155 (also known as poliovirus receptor, PVR) and CD112 (Nectin-2) are shared ligands for CD226 and the inhibitory T-cell immunoreceptor with Ig and ITIM domains (TIGIT). A substantial proportion of preceding studies were confined to the role of CD226 in immune cells such as T cells and NK cells. It is well-documented that the primary function of the regulatory mechanism being studied is to control the differentiation process and effector function of naive T cells and NK cells [13,14,15]. Recent studies have shown that CD226 is expressed on monocytes and macrophages [16,17]. It has been hypothesised that the phenomenon may exert a significant influence on the polarisation of macrophages and the migration of monocytes [18,19,20,21,22]. Moreover, Kakehi et al. have demonstrated that the extracellular domain of DNAM-1 functions to inhibit the formation of multinucleated osteoclasts through its binding to the corresponding ligand, PVR [23]. However, the role of CD226 in osteoclasts during periodontitis has yet to be clarified.

In this study, we highlight CD226 as a critical immune checkpoint in osteoimmunology. Although historically investigated for its roles in NK- and T-cell-mediated cytotoxicity against tumours and viral infections, CD226 has been implicated in pathological bone remodelling and inflammatory cell recruitment during periodontal tissue destruction. Situated at the interface between inflammation and bone biology, CD226 links bacterial-triggered immune activation to osteoclast function, thereby directly contributing to alveolar bone resorption. Given this central functional role, targeting CD226 may attenuate inflammatory osteoclastogenesis and mitigate bone loss, opening new avenues for host-modulation therapies in regenerative periodontology.

## 2. Materials and Methods

### 2.1. Experimental Murine Periodontitis Model

Six- to eight-week-old male C57BL/6 mice were obtained from the Air Force Medical University animal centre. Dr Marco Colonna kindly gifted us with C57BL/6-background CD226 knockout (CD226-KO) mice. All experiments were carried out following protocols that had been approved by the Animal Experimentation and Ethics Committee of Air Force Medical University. The periodontal disease model was induced as previously described [24]. Briefly, the mice were anaesthetised via intraperitoneal injection of pentobarbital sodium. The tip of a pre-bent C+ nickel–titanium root canal file was slowly filed into the interproximal space between the maxillary molars. Then, 0.20 mm wide, 3–5 mm long orthodontic ligatures were inserted. Mice in the sham control group were subjected to the identical surgical procedure, except that no wire was placed. Ten days following the induction of periodontitis, the mice were euthanised using an overdose of isoflurane.

### 2.2. Micro-CT Scanning and Reconstruction

Maxillary bones were harvested immediately after euthanasia. Surrounding soft tissues were meticulously excised, and samples were briefly washed using phosphate-buffered saline (PBS). Tissues were then fixed in 4% paraformaldehyde (PFA) at 4 °C for 24 h and subsequently kept in 75% ethanol until high-resolution microCT scanning (Yxlon, Hamburg, Germany). The raw data were exported in order to reconstruct three-dimensional images, and each scan was reoriented to the same position in order to analyse bone loss using anatomical landmarks. As previously described [24], the cement–enamel junction (CEJ)–alveolar bone crest (ABC) distance was measured at six predefined locations on both the buccal and palatal surfaces.

### 2.3. Histology

Mouse maxillae were collected from mice and fixed in 4% PFA for histological examination. Subsequently, the samples were decalcified in 10% EDTA for 20 days, followed by dehydration and embedding in paraffin. The fixed tissues were cut into 5 μm thick sections for subsequent histological staining and TRAP staining.

### 2.4. TRAP Staining and TRAP-Activity Measurement

Differentiated osteoclasts and tissue sections were subjected to tartrate-resistant acid phosphatase (TRAP) staining using commercial TRAP staining kits following the manufacturer’s instructions. TRAP-positive multinucleated cells with ≥3 nuclei were identified under light microscopy and defined as mature osteoclasts. Three to five random visual fields covering the alveolar bone area were captured for each sample. Cell-culture supernatants from osteoclast cultures were collected for quantitative TRAP-activity detection with corresponding assay kits.

### 2.5. Osteoclast Differentiation

Primary bone marrow cells were isolated from the femur and tibia bone marrow of 8-week-old mice. The cells were cultured in complete Minimum Essential Medium α (α-MEM) containing 10% foetal bovine serum (FBS), 1% penicillin/streptomycin, and 25 ng/mL macrophage colony-stimulating factor (M-CSF) for 24 h, after which the non-adherent cells were collected and cultured in fresh medium supplemented with 50 ng/mL receptor activator of nuclear factor-κB ligand (RANKL) and 25 ng/mL M-CSF; every two days, the cell culture medium was changed, and this was continued until mature osteoclasts developed.

### 2.6. Cell Isolation

Gingival cells were isolated as previously described [25]. In brief, gingival tissue from the upper and lower jaws was carefully dissociated. Connective tissues attached to gingiva were trimmed off, and gingival tissues were collected for subsequent enzymatic digestion involving collagenase type IV (0.5 mg/mL) for one hour at 37 °C. The cells were then washed and collected by centrifugation before being resuspended in Dulbecco’s modified Eagle medium (DMEM). Peritoneal macrophages were obtained via lavage of the peritoneal cavity using 20 mL of PBS. The macrophages were resuspended in 10% FBS-supplemented DMEM and seeded into 24-well plates. Four hours post-seeding, the plates were washed with PBS to remove non-adherent cells, after which the adherent macrophages were collected for further analysis.

### 2.7. Flow Cytometric Analysis

Peripheral blood from mice was obtained by cardiac puncture, followed by lysis of red blood cells using a red cell lysis buffer. Gingival cells, peritoneal macrophages, and peripheral blood cells were incubated with different fluorochrome-labelled antibodies at 4 °C for 30 min. The following antibodies were then employed to determine the cell phenotypes: anti-CD226-APC, anti-F4/80-FITC, anti-Ly6C-PE/CY7, and anti-CD11b-PE. All antibodies were obtained from BioLegend (San Diego, CA, USA). All experiments were performed three independent times, and representative results are presented in the corresponding figures.

### 2.8. qRT-PCR

TRIzol reagent (Life Technologies, Carlsbad, CA, USA) was applied to extract total RNA from cultured cells and isolated periodontal tissue specimens. For cDNA synthesis, 2 μg of purified RNA was reverse-transcribed with PrimeScript RT Master Mix (Yeasen, Shanghai, China). Real-time quantitative PCR reactions were run on the CFX96 platform employing SYBR Premix Ex Taq II (Yeasen, China). All assays were performed in accordance with the manufacturer’s instructions. The relative amount of target mRNA (CD226, TIGIT, CD155, CD112, Acp5, Nfatc1, Ctsk, c-Fos, IL-1β, TNF-α, and IL-6) was determined using the 2^−ΔΔCt^ method and normalised to the 18S housekeeping gene. The qRT-PCR amplification was performed under the following thermal profile: an initial 3 min denaturation step at 95 °C, then 40 repetitive cycles consisting of 10 s denaturation at 95 °C and 30 s annealing at 60 °C. Each sample was assayed in technical triplicate.

### 2.9. Statistical Analysis

Quantitative data are expressed as the mean ± standard deviation (SD), and statistical analyses were implemented in GraphPad Prism 8. The Shapiro–Wilk test was applied to evaluate whether datasets followed a normal distribution. Two-group comparisons were conducted using a two-tailed Student’s *t*-test. For multiple-group comparisons, one-way ANOVA was adopted, followed by Tukey’s post hoc test. Statistical significance was defined as *p* < 0.05.

## 3. Results

### 3.1. CD226 Knockout Reduced Alveolar Bone Loss in an Experimental Murine Periodontitis Model

**Figure 1:**
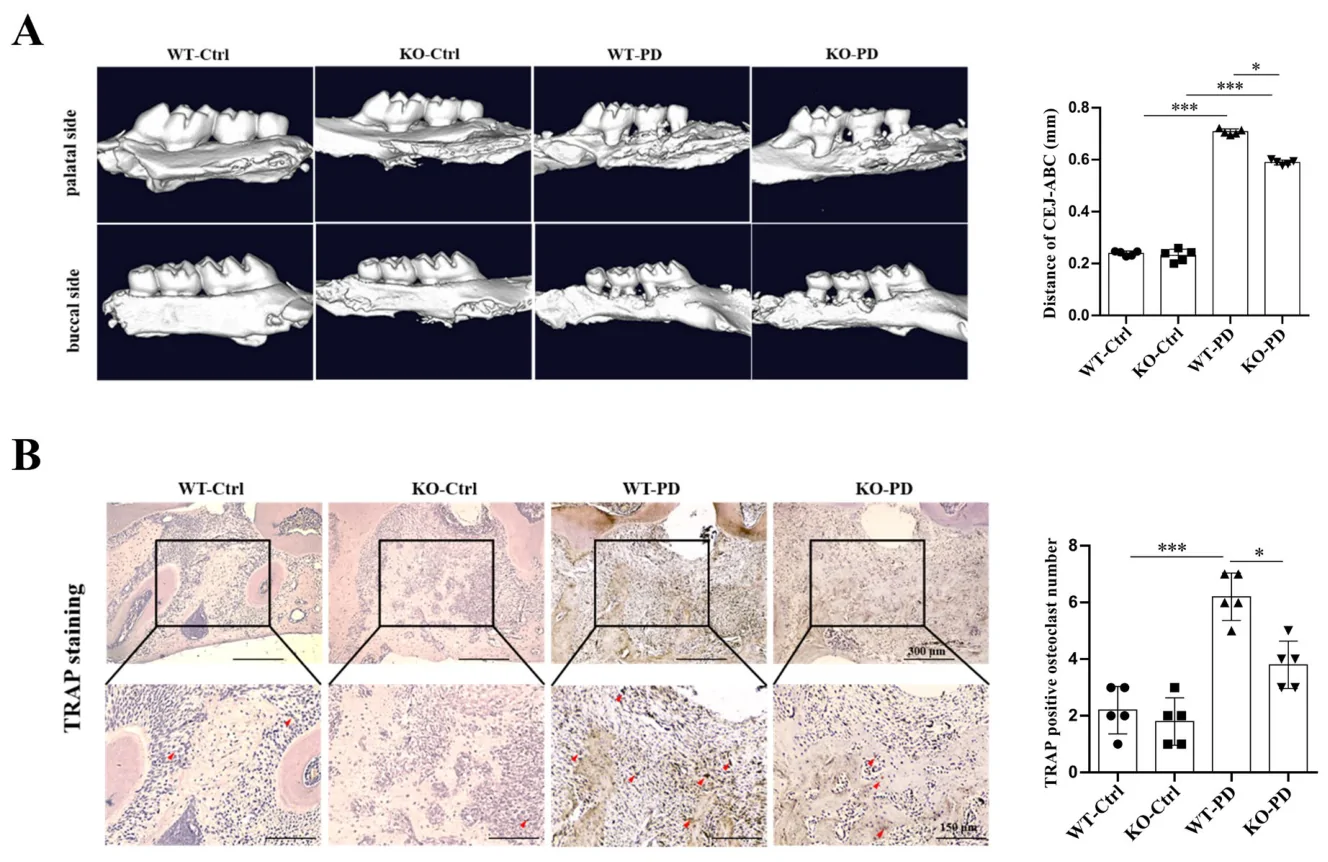
The knockout of CD226 has been shown to attenuate alveolar bone resorption in periodontitis mice: (**A**) Micro-CT was used to generate 3D-reconstructed images of maxillae obtained from the WT and CD226-KO groups (*n* = 5). Measurements of the average ABC-to-CEJ distance were taken at three positions (mesial, central, and distal sites), and the results are shown as mean ± SD. Ctrl stands for the sham-operated control group, and PD represents the ligature-induced periodontitis model group. (**B**) TRAP-stained osteoclasts (marked with red arrows) in periodontium sections of each group. Microscopic counting was performed to determine the number of TRAP-stained osteoclasts per group, and data are expressed as mean ± SD. * *p* < 0.05, *** *p* < 0.001.

To determine how CD226 affects the process of bone destruction, with a particular focus on osteoclastogenesis in the context of periodontitis, the present study employs a CD226 knockout mouse model as an experimental subject. As demonstrated in Figure 1A, the specimens from the WT periodontitis group (WT-PD) exhibited substantial bone loss, as evidenced by the assessment of CEJ-ABC distances on the palatal and buccal surfaces. When compared to the WT periodontitis group (WT-PD), alveolar bone resorption was significantly reduced in CD226-KO mice with periodontitis (KO-PD), and these mice showed a stronger osteoprotective effect. Furthermore, the samples of alveolar bone were harvested for TRAP staining. Histomorphometric analysis showed that the number of TRAP-positive cells was significantly reduced in CD226-KO mice with periodontitis (KO-PD) (Figure 1B).

### 3.2. CD226 Expression Was Detected in Both Osteoclasts and Their Precursor Cells

**Figure 2:**
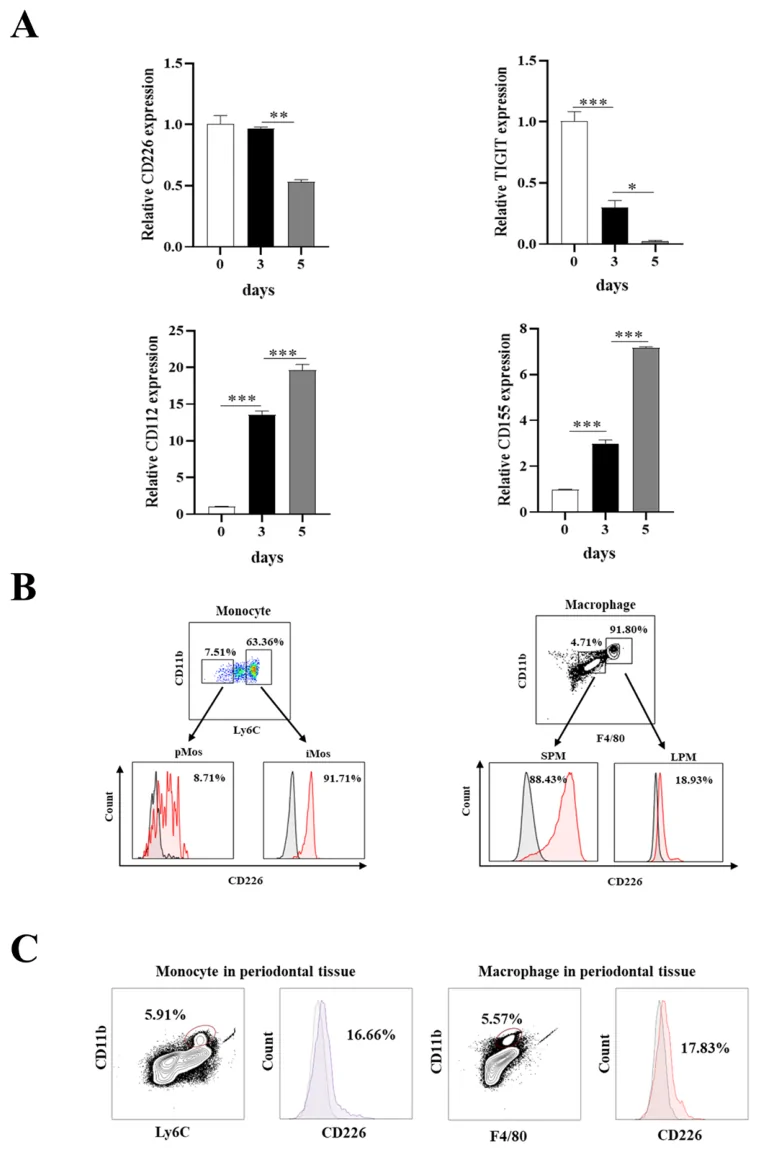
The expression of CD226 is distinct among various monocyte/macrophage subsets and osteoclasts during the process of osteoclastogenesis: (**A**) The expression of mRNA for CD226, TIGIT, CD112, and CD155 was observed on osteoclasts during the process of osteoclastogenesis. (**B**) The left panel shows the expression of CD226 on blood inflammatory monocytes (iMos, defined as CD11b^+^Ly6C^+^) and patrolling monocytes (pMos, defined as CD11b^+^Ly6C^−^). The right panel demonstrates CD226 expression on small peritoneal macrophages (SPMs, CD11b^+^F4/80^lo^) and large peritoneal macrophages (LPMs, CD11b^hi^F4/80^hi^). (**C**) CD226 expression on monocytes (CD11b^+^Ly6C^+^, left) and macrophages (CD11b^+^F4/80^+^, right) in periodontal tissues is presented. * *p* < 0.05, ** *p* < 0.01, *** *p* < 0.001.

The following study examined the expression of CD226 in osteoclasts and their monocyte/macrophage precursors. In vitro osteoclast differentiation was induced from primary bone marrow cells in the presence of RANKL and M-CSF. The expression levels of CD226 and other related molecules (TIGIT, CD155, and CD112) were observed to change during the process. The results demonstrated a gradual downregulation of CD226 and TIGIT expression during the process of osteoclastogenesis. However, the expression of their ligands, CD112 and CD155, increased during the differentiation process (Figure 2A).

It is hypothesised that monocytes and macrophages are precursors of osteoclasts. CD226 expression was found to be elevated on inflammatory monocytes (iMos) and small peritoneal macrophages (SPMs), a finding that aligns with the conclusions of preceding studies (Figure 2B). However, as demonstrated in Figure 2C, a modest percentage (approximately 20%) of monocytes and macrophages in mouse periodontal tissues expressed CD226.

### 3.3. Knockout of CD226 Inhibited Osteoclastogenesis In Vitro

**Figure 3:**
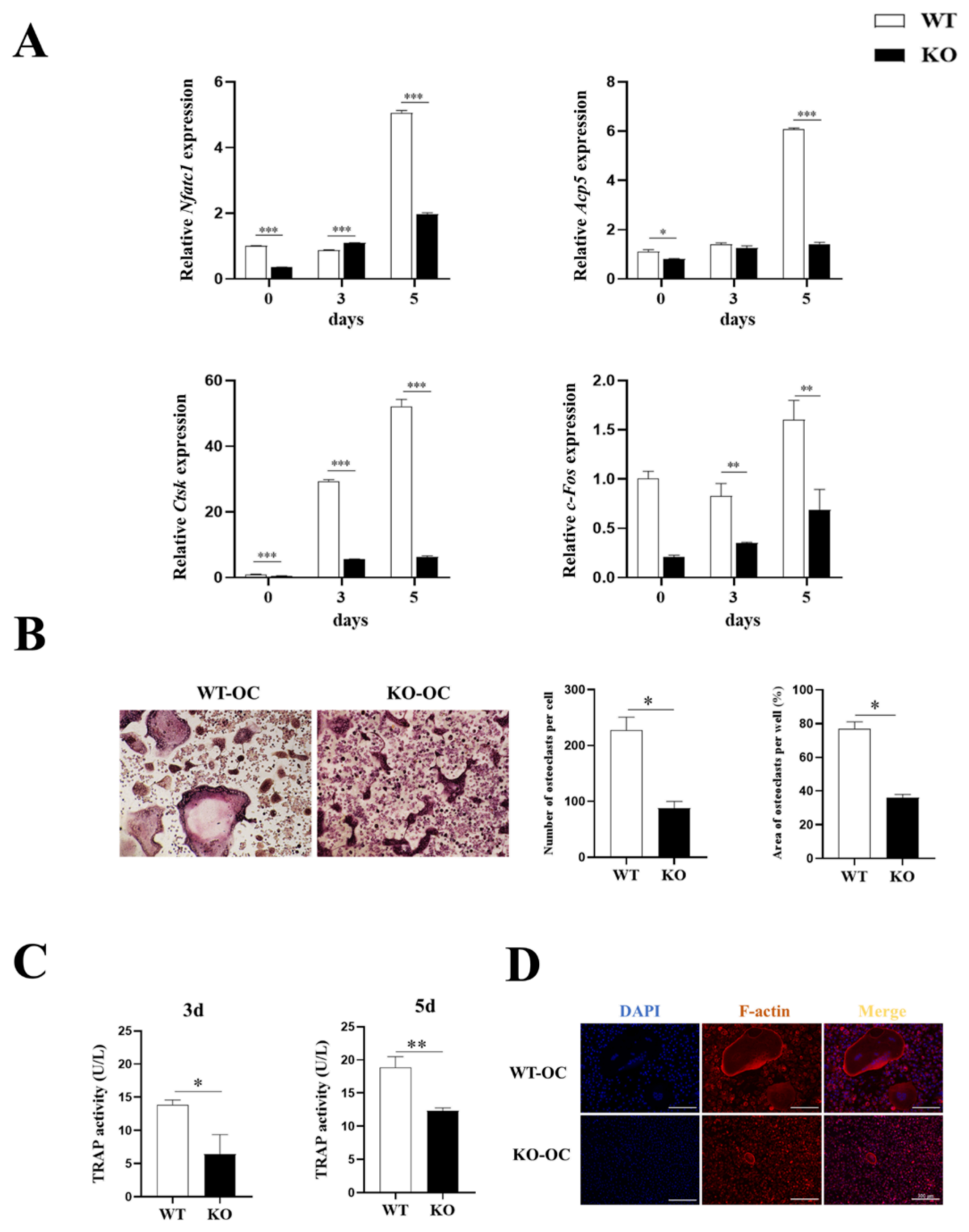
The knockout of CD226 has been demonstrated to inhibit osteoclastogenesis in vitro: (**A**) The present study employed qRT-PCR analysis to investigate the expression of osteoclast formation-specific genes in osteoclasts induced by primary bone marrow cells from WT and CD226-KO groups. The x-axis indicates days after osteoclast precursor stimulation with RANKL and M-CSF. (**B**) TRAP staining was implemented, and the count of TRAP-positive cells along with their area was determined. (**C**) TRAP activity assessment using cell culture medium was accomplished on the 3rd and 5th days during the process of osteoclastogenesis. (**D**) Representative immunofluorescent images of F-actin-stained osteoclasts from WT and CD226-KO mice. * *p* < 0.05, ** *p* < 0.01, *** *p* < 0.001. The data are representative of three independent experiments.

We next investigated how CD226 deficiency affects osteoclast differentiation in vitro. After isolating osteoclast precursors from the bone marrow of CD226-KO mice and control mice, the cells were cultured in the presence of RANKL and M-CSF for 5 days. As shown in Figure 3A, the expression levels of multiple osteoclastogenic-related genes, including *acid phosphatase 5* (*Acp5*), *nuclear factor of activated T-cells cytoplasmic 1* (*Nfatc1*), *cathepsin K* (*Ctsk*), and *cellular oncogene* (*c-Fos*), were lower in CD226-KO mice. In addition, we observed a significant reduction in both osteoclast numbers and osteoclast surface areas in CD226-KO mice when compared with control mice (Figure 3B). Consistent with the findings from TRAP staining, the knockout of CD226 further inhibited osteoclast TRAP activity (Figure 3C). F-actin rings are essential for the bone-resorbing function of osteoclasts. We observed that CD226 knockout significantly decreased the number of F-actin rings (Figure 3D). Collectively, these findings suggested that CD226 deficiency was capable of inhibiting osteoclastogenesis in vitro.

### 3.4. Loss of CD226 Resulted in Decreased Inflammation in Periodontitis Mice

**Figure 4:**
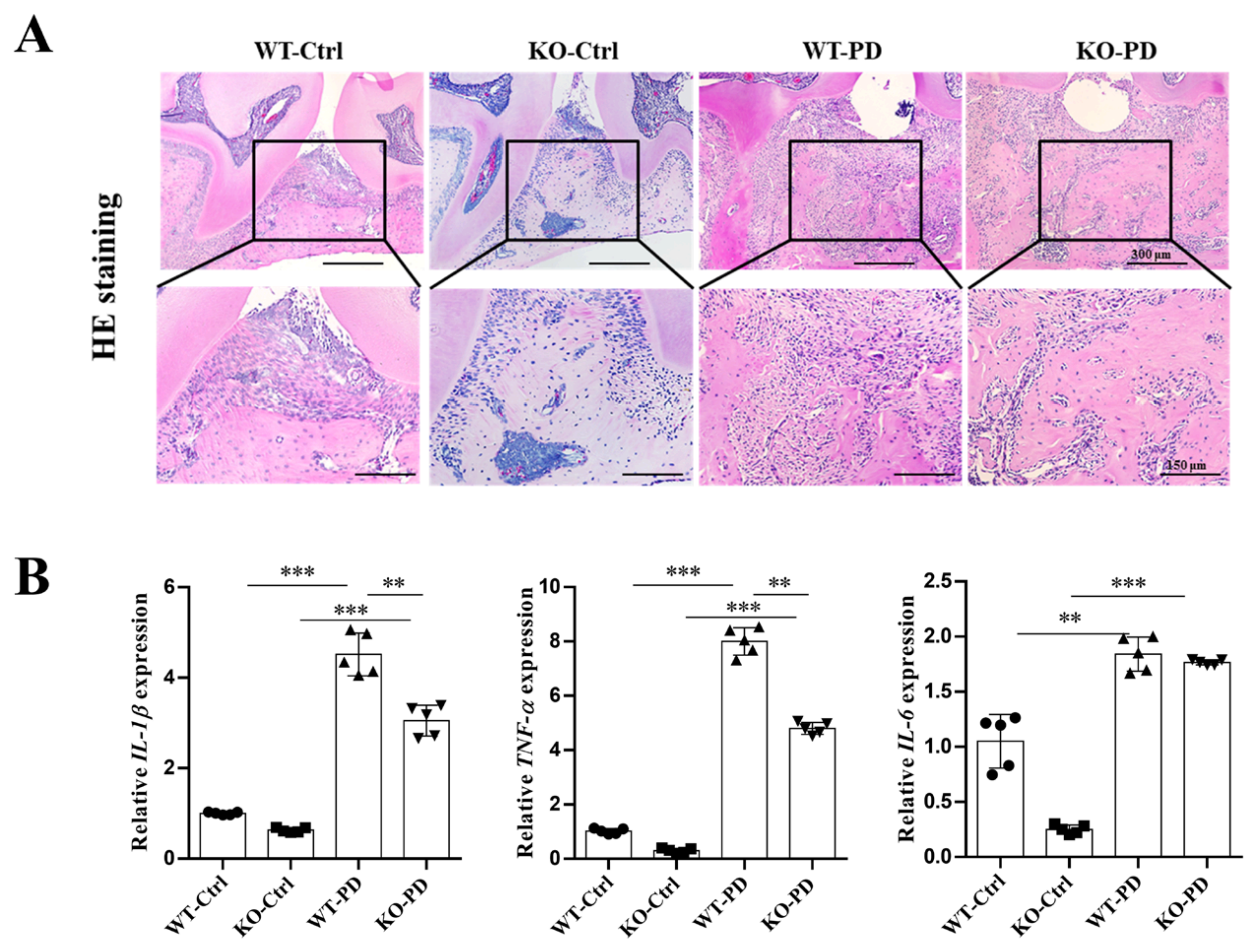
The knockout of CD226 has been shown to ameliorate ligature-induced inflammation in periodontal tissues: (**A**) Histological analysis of H&E staining in the periodontium of WT and CD226-KO periodontitis mice (*n* = 5). (**B**) qRT-PCR was employed to assess the mRNA levels of the inflammatory cytokines IL-1β, TNF-α, and IL-6 in the periodontal tissue of WT and CD226-KO mice with periodontitis. ** *p* < 0.01, *** *p* < 0.001.

Periodontitis is an inflammatory disease in which cytokines in periodontal tissues can induce osteoclast differentiation and activation. To examine the role of CD226 in periodontitis, we used a ligature-induced mouse model. Histological analysis of periodontal tissues showed reduced bone resorption and fewer infiltrating inflammatory cells in CD226-KO mice with periodontitis compared with WT periodontitis mice (Figure 4A). We further analysed inflammation-related cytokines in periodontal tissues and found that CD226 knockout significantly decreased the expression of the proinflammatory cytokines IL-1β and TNF-α (Figure 4B). Collectively, these results suggested that CD226 is important in the development of ligature-induced mouse periodontitis.

### 3.5. CD226 Knockout Changed the Distribution of Monocytes/Macrophages in Mice with Periodontitis

**Figure 5:**
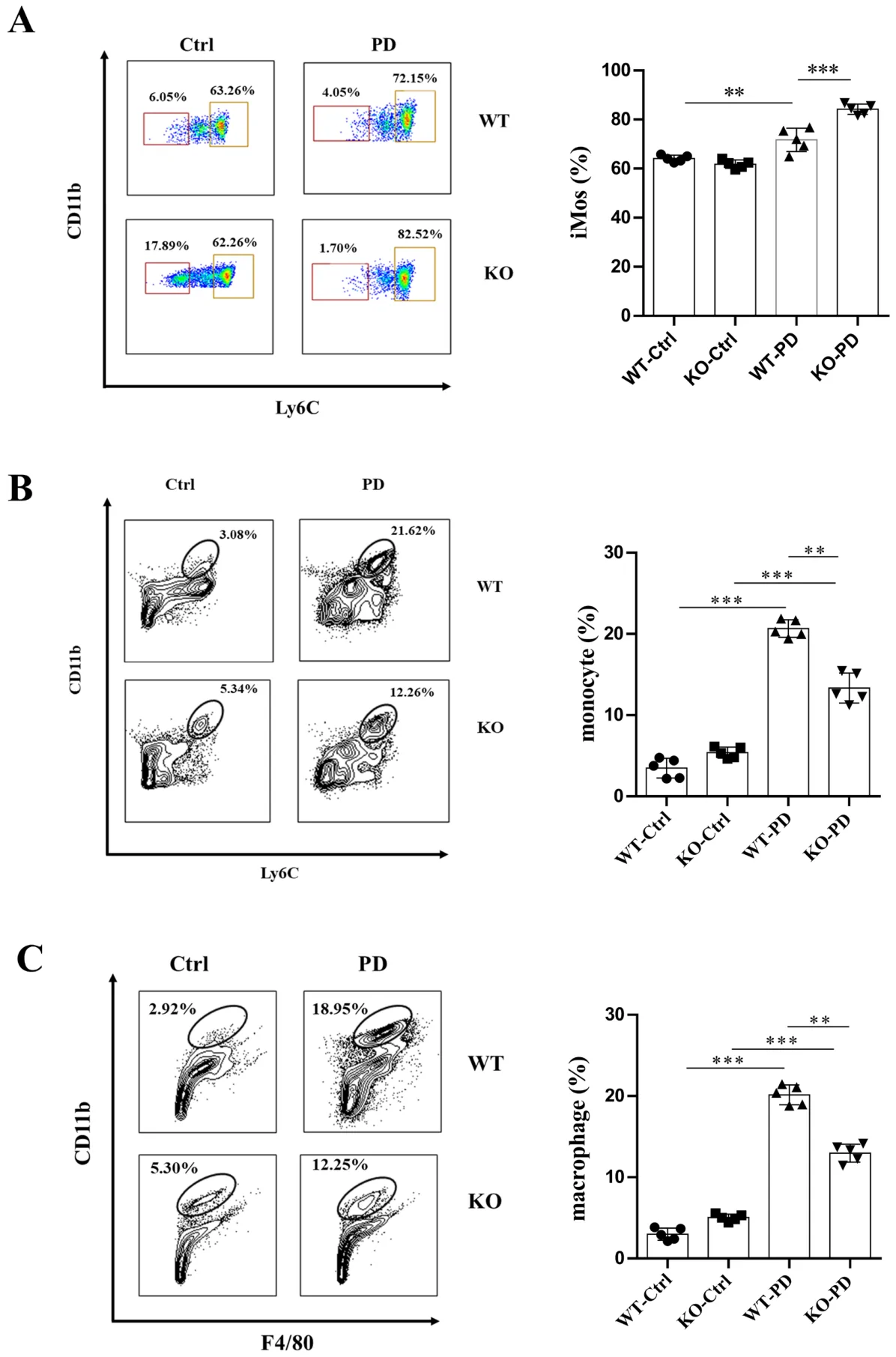
CD226 deletion has been demonstrated to have an impact on the monocyte and macrophage distribution: (**A**) The percentage of different blood monocyte subsets in WT and CD226-KO periodontitis mice (*n* = 5). (**B**) The percentage of periodontal monocytes in WT and CD226-KO periodontitis mice (*n* = 5). (**C**) The percentage of periodontal macrophages in WT and CD226-KO periodontitis mice. ** *p* < 0.01, *** *p* < 0.001.

Monocytes are of significance as a source of macrophages, and both monocytes and macrophages are precursors of osteoclasts. It has been demonstrated that the deletion of CD226 has the capacity to suppress the process of M1 polarisation in macrophages, thereby inhibiting inflammatory responses. The aim of this study was to determine whether CD226 deletion could alter the balance between monocytes and macrophages in a mouse model of periodontitis. As demonstrated in the present study, knocking out CD226 resulted in a significant increase in the proportion of peripheral blood iMos in periodontitis mice (Figure 5A). However, the knockout of CD226 resulted in a significant decrease in the percentage of monocytes and macrophages in the periodontal tissues of murine periodontitis models (Figure 5B,C).

## 4. Discussion

Periodontal disease is widely recognized as a chronic infectious-inflammatory disorder. Severe periodontitis leads to the destruction of connective tissue attachment, progressive alveolar bone loss, and ultimately tooth loss. Among these factors, the loss of bone tissue induced by periodontitis is primarily attributable to the dysfunction of osteoclasts and their precursors [1,2,4,26]. Consequently, the regulation of osteoclast differentiation and function may offer a potential therapeutic approach for addressing periodontal bone loss.

In this study, the CD226-KO mouse periodontitis model was utilised to observe alterations in bone mass. The results of the quantitative Micro-CT analysis showed that the knockout of CD226 led to a significant alleviation of bone resorption and a reduction in osteoclast numbers in periodontitis mice. However, the expression and effects of CD226 in osteoclastogenesis were not well understood. The results demonstrated that CD226 expression did not show any significant variation during the first three days of osteoclastogenesis. A marked decrease in CD226 mRNA levels was observed on day 5 of differentiation. Furthermore, the expression of TIGIT, which could compete with CD226 for binding to CD155, also decreased. The results demonstrated that following exposure to RANKL, there was a substantial enhancement in the expression of CD155 and CD112, which function as ligands for CD226, from day 3 to day 5. These results prompted us to verify the role of CD226 in the differentiation of osteoclasts.

To determine how CD226 regulates osteoclast differentiation, primary bone marrow cells isolated from CD226-KO and WT mice were cultured with M-CSF and RANKL. This study aimed to investigate the expression levels of NFATc1, ACP5, CTSK, and C-FOS in CD226-KO mice. The obtained results demonstrated a decrease in the expression of these markers and transcription factors, which play important roles in osteoclast differentiation and function. Furthermore, TRAP and F-actin staining revealed that the knockout of CD226 inhibited osteoclastogenesis. As demonstrated in previous studies, the binding of CD155 to the extracellular domain of CD226 has been shown to suppress the formation of multinucleated osteoclasts [23]. These results suggest that CD226 and its ligand CD155 may have opposite functions in osteoclastogenesis. CD226 has been demonstrated to deliver stimulatory signals to effector cells; conversely, CD155 has been shown to mediate direct inhibitory signalling. The combined effect of the CD226-CD155 interaction is vital for the development and homeostasis of osteoclasts.

As demonstrated in the extant literature, macrophages and monocytes are the origins of osteoclasts. Moreover, these cells are associated with bone resorption in periodontitis [8,9,10,27,28,29]. Macrophages of the M1 phenotype have been observed to express elevated levels of proinflammatory cytokines, with the capacity to induce osteoclast differentiation [29,30]. In the event of M1 polarisation being blocked, M2 macrophages have been shown to exert anti-inflammatory effects in periodontal tissue. CD226 has been reported to promote the M1-type macrophages’ polarisation and to inhibit the M2-type polarisation [19]. In this study, it was found that there was less inflammatory cell infiltration in the periodontal tissues of CD226-KO periodontitis mice than in the control group. Furthermore, it was found that proinflammatory cytokines such as IL-1β and TNF-α were significantly reduced in CD226-KO periodontitis mice. These findings were consistent with the results described above.

Following the entry of monocytes into specific tissues, a process of differentiation into macrophages or osteoclasts is initiated in response to specific stimulants. It has been reported that the interaction between CD226 and its ligand, CD155, drives monocyte migration [16,18]. In the present study, CD226 knockout was found to alter monocyte distribution in a murine periodontitis model, with the CD226-KO group showing a significant increase in the percentage of monocytes in peripheral blood compared to the control group. Subsequent analysis indicated a decline in the percentage of macrophages within the periodontal tissues. The present findings are consistent with those of previous research [31], which indicated that CD226 may mitigate inflammatory responses and bone damage by modulating the migration and distribution of monocytes.

Despite the identification of CD226 as a potential regulator of osteoclasts and their progenitors with regard to the regulation of bone metabolism, the current study has several limitations. Firstly, the molecular mechanism underlying CD226-mediated regulation of osteoclast differentiation has not yet been clarified. Secondly, in the context of animal studies, CD226 has been observed to exert divergent functional effects on T cells and other immune cells. It is not possible to exclude the occurrence of these effects on bone resorption in cases of periodontitis. Consequently, further research is required into CD226, specifically expressed on osteoclasts and their progenitors. Thirdly, only male mice were used for all in vivo experiments in this work. Thus, potential sex-dependent differences in CD226-driven periodontal bone destruction cannot be ruled out, and future investigations incorporating both male and female animals are warranted. Furthermore, we cannot completely rule out potential alterations in osteoblast activity in CD226-KO mice, which may also contribute to alveolar bone phenotypes. Further investigations focusing on osteoblast-related phenotypes are currently ongoing in our laboratory.

## 5. Conclusions

In summary, the present research proposes a model that describes how CD226 functions in osteoclasts and their progenitors as follows: CD226 has been identified as being expressed in osteoclasts and their progenitors, and it has been demonstrated to have intrinsic functions in periodontitis-induced bone loss. CD226 deficiency has been demonstrated to inhibit osteoclastogenesis in vitro whilst concomitantly modifying the distribution of monocytes and macrophages within periodontal tissues. In a murine periodontitis model, the knockout of CD226 has been shown to decrease inflammatory response and alleviate bone damage in vivo. Consequently, the targeting of CD226 in osteoclasts and their progenitors has the potential to be developed into a potent therapy for periodontitis-related bone loss.

## Data Availability

The authors confirm that the data underlying the findings of this study can be found within the article.
